# Inflation versus projection sets in aperiodic systems: the role of the window in averaging and diffraction

**DOI:** 10.1107/S2053273320007421

**Published:** 2020-07-09

**Authors:** Michael Baake, Uwe Grimm

**Affiliations:** a Fakultät für Mathematik, Universität Bielefeld, Postfach 100131, 33501 Bielefeld, Germany; b School of Mathematics and Statistics, The Open University, Walton Hall, Milton Keynes MK7 6AA, UK; c School of Natural Sciences, University of Tasmania, Private Bag 37, Hobart TAS 7001, Australia

**Keywords:** quasicrystals, projection method, inflation rules, diffraction, hyperuniformity

## Abstract

Tilings based on the cut-and-project method are key model systems for the description of aperiodic solids. Typically, quantities of interest in crystallography involve averaging over large patches, and are well defined only in the infinite-volume limit. In particular, this is the case for autocorrelation and diffraction measures. For cut-and-project systems, the averaging can conveniently be transferred to internal space, which means dealing with the corresponding windows. In this topical review, this is illustrated by the example of averaged shelling numbers for the Fibonacci tiling, and the standard approach to the diffraction for this example is recapitulated. Further, recent developments are discussed for cut-and-project structures with an inflation symmetry, which are based on an internal counterpart of the renormalization cocycle. Finally, a brief review is given of the notion of hyperuniformity, which has recently gained popularity, and its application to aperiodic structures.

## Abstract 2

Averaged quantities such as mean shelling numbers, scaling behaviour or diffraction for cut-and-project sets can conveniently be computed in internal space, also for systems with fractally bounded windows.

## 1. Introduction   

The discovery of quasicrystals in the early 1980s (Shechtman *et al.*, 1984 ▸) not only led to a reconsideration of the fundamental concept of a crystal [see Grimm (2015 ▸) and references therein], but also highlighted the need for a mathematically robust treatment of the diffraction of systems that exhibit aperiodic order. The foundations for a rigorous approach were laid by Hof (1995 ▸). In particular, the measure-theoretic approach via the autocorrelation and diffraction measures allows for a mathematically rigorous discussion and separation of the different spectral components, the pure point, singular continuous and absolutely continuous parts; see Baake & Grimm (2012 ▸) for background and examples, and ch. 9 in Baake & Grimm (2013 ▸) for a systematic exposition. For general background on the theory of aperiodic order, we refer readers to Pytheas Fogg (2002 ▸), Allouche & Shallit (2003 ▸), Queffélec (2010 ▸), Baake & Grimm (2013 ▸), Kellendonk *et al.* (2015 ▸), Akiyama & Arnoux (2020 ▸) and references therein.

Within a few years, it was established that regular model sets (Moody, 2000 ▸), meaning systems obtained by projection from higher-dimensional lattices via cut-and-project mechanisms with ‘nice’ windows, have pure point diffraction (Schlottmann, 2000 ▸; Richard & Strungaru, 2017*a* ▸). We refer readers to the discussion in Baake & Grimm (2013 ▸) for details and examples, and to Baake *et al.* (2016 ▸) for an instructive application of the cut-and-project approach to an experimentally observed structure with 12-fold symmetry. The result on the pure point nature of diffraction holds for rather general setups, including cut-and-project schemes with non-Euclidean internal spaces. It has recently been generalized to weak model sets of extremal densities (Baake *et al.*, 2017 ▸; Richard & Strungaru, 2017*b* ▸), for which the window may even entirely consist of boundary, that is, has no interior; see also Strungaru (2017 ▸, 2020 ▸) for recent work on pure point spectra.

While systems based on a cut-and-project scheme are generally well understood, this is less so for systems originating from substitution or inflation rules, which constitute another popular method of generating systems with aperiodic order; see Queffélec (2010 ▸), Baake & Grimm (2013 ▸), Frettlöh (2017 ▸), and references therein for details. There has been recent progress particularly on substitutions of constant length; see Mañibo (2017 ▸), Bartlett (2018 ▸), Berlinkov & Solomyak (2019 ▸), Baake *et al.* (2020 ▸), Baake, Frank *et al.* (2019 ▸), Bufetov & Solomyak (2020 ▸).

There are familiar examples of inflation-based structures for all spectral types, such as the Fibonacci chain for a pure point diffractive system, the Thue–Morse chain for a system with purely singular continuous diffraction, and the binary Rudin–Shapiro chain as the paradigm of a system with absolutely continuous diffraction; see Pytheas Fogg (2002 ▸), Allouche & Shallit (2003 ▸), Baake & Grimm (2013 ▸) for details. When one equips the Rudin–Shapiro chain with balanced weights (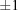), it becomes homometric with the binary Bernoulli chain with random weights 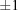 (Baake & Grimm, 2009 ▸). It is easy to construct inflation-based systems which combine any of these spectral components in their diffraction; see Baake *et al.* (2013 ▸) for examples. As of today, the celebrated Pisot substitution conjecture (which stipulates that an irreducible Pisot substitution has a pure point spectrum) remains open; see Akiyama *et al.* (2015 ▸) for a review of the state of affairs.

While diffraction was the first property to be analysed in detail, many other questions from traditional crystallography and lattice theory require an extension to their aperiodic counterparts (Baake & Zeiner, 2017 ▸). In particular, classic counting problems based on lattices, when reformulated for point sets in aperiodic tilings, need both a conceptual reformulation and new tools to tackle them. The key observation is the necessity to employ averaging concepts, and then tools from dynamical systems and ergodic theory (Queffélec, 2010 ▸; Solomyak, 1997 ▸; Baake & Grimm, 2013 ▸). If one is in the favourable situation of point sets that emerge from either the projection formalism or an inflation procedure, many of these averaged quantities are well defined and can actually be calculated; see Baake & Grimm (2003 ▸) and references therein. Despite good progress, many questions in this context remain open.

Let us sketch how this introductory review is organized. Our guiding example in this exposition is the classic self-similar Fibonacci tiling of the real line. Its descriptions as an inflation set and as a cut-and-project set are reviewed in Section 2. As a simple example of the role of the window in averaging, we discuss the averaged shelling for this system in Section 3. This is followed by a brief review of the standard approach to diffraction in Section 4, where we exploit the description of the Fibonacci point set as a cut-and-project set and the general results for the diffraction of regular model sets.

In Section 5, we recapitulate the recently developed internal cocycle approach. For systems which possess both an inflation and a projection interpretation, such as the Fibonacci tiling, the inflation cocycle can be lifted to internal space. This makes it possible to efficiently compute the diffraction of certain cut-and-project systems with complicated windows, such as windows with fractal boundaries, as are commonly found in inflation structures. To explore this further, we reconsider planar examples, based on the Fibonacci substitution, in Section 6.

Finally, in Section 7, we discuss the use of ‘hyperuniformity’ as a measure of order in Fibonacci systems. This amounts to an investigation of the asymptotic behaviour of the total diffraction intensity near the origin. It turns out that this can dinstinguish between generic and inflation-invariant choices for the window in the cut-and-project scheme.

## 2. The Fibonacci tiling revisited   

Let us start with a paradigm of aperiodic order in one dimension, the classic Fibonacci tiling. It can be defined via the primitive two-letter inflation rule
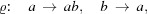
where *a* and *b* represent *tiles* (or intervals) of length τ = 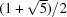 and 1, respectively. The corresponding incidence matrix is given by
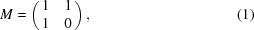
which has the Perron–Frobenius eigenvalue τ. Its left and right eigenvectors read
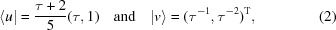
where we employ Dirac’s intuitive ‘bra-c-ket’ notation, which makes it easy to distinguish row and column vectors. We normalize the right eigenvector 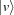 such that 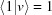, which means that its entries are the *relative frequencies* of the tiles. For later convenience, we normalize the left eigenvector 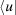 by setting 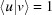, rather than using the vector of natural tile lengths itself. With this normalization, we have
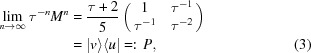
where 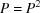 is a symmetric projector of rank 1 with spectrum 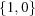.

Starting from the legal seed 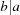, where the vertical bar denotes the origin, and iterating the square of the inflation rule 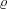 generates a tiling of the real line that is invariant under 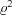; see Example 4.6 in Baake & Grimm (2013 ▸) for details and why it does not matter which of the two fixed points of 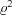 one chooses. Let us use the left endpoints of each interval as *control points* and denote the set of these points by 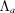 and 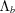, respectively. Clearly, since 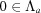 and all tiles have either length τ or length 1, all coordinates are integer linear combinations of these two tile lengths, and we have
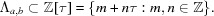
The incidence matrix *M* only contains information about the number of tiles under inflation, not about their positions. To capture the latter, and thus encode the full information of the inflation, we consider the set-valued *displacement matrix*

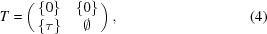
where 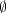 denotes the empty set. Note that *T* is the geometric counterpart of the instruction matrices that are used in the symbolic context (Queffélec, 2010 ▸). The matrix elements of *T* are sets that specify the relative displacement for all tiles under inflation. For instance, the two entries in the first column correspond to a long tile with relative shift 0 and a small tile with shift τ originating from inflating a long tile. Clearly, the inflation matrix *M* is recovered if one takes the elementwise cardinality of *T*, noting that the empty set has cardinality 0.

The inflation rule 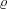 induces an iteration on pairs of point sets, namely
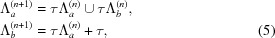
with suitable initial conditions 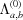. When one starts with the left endpoints of a legal seed, this iteration precisely reproduces the endpoints of the corresponding successive inflation steps. In this case, the union on the right-hand side is disjoint. In particular, for the above choice of 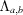, one needs 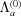 = {0} and 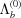 = {−1}.

The point sets 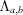 also have an interpretation as a cut-and-project set. Here, we use the natural (Minkowski) embedding of the module 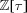 in the plane 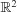, by associating to each 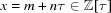 its image 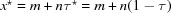 under algebraic conjugation (which maps 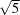 to 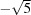). This gives
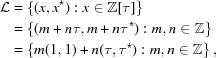
which is a planar lattice with basis vectors 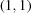 and 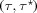; see Baake & Grimm (2013 ▸) and Baake *et al.* (2016 ▸) for details and further examples. Here, we refer to the two one-dimensional subspaces of 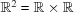 as the *physical* and the *internal* space, respectively. The physical space hosts our point sets 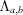, while the windows are subsets of the internal space, with the 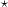-map providing the relevant link between the two spaces.

**Figure 1:**
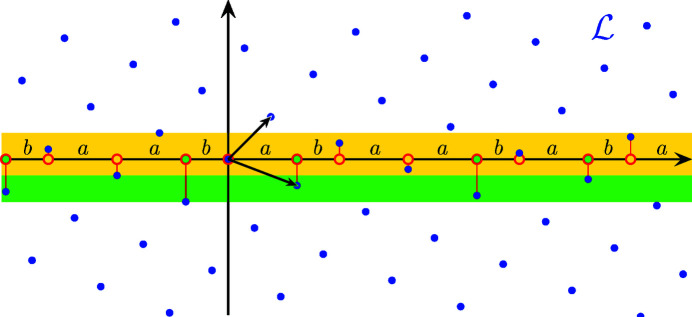
Cut-and-project description of the Fibonacci chain from the lattice 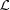 (blue dots). The windows 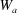 and 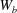 are the cross sections of the yellow and green strips, respectively.

The two point sets 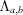 are given by the projection of all points of 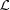 within two strips; see Fig. 1 ▸. These strips are defined by their cross sections, usually called *windows*, which are the half-open intervals 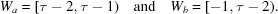
With 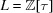, the projection of 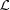 into physical space, the point sets are thus given by
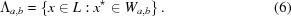
One of the powerful properties of the cut-and-project approach is that we can switch between the physical space and the internal space, and calculate properties in the latter. Taking the 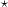-image of (5), we obtain the relations
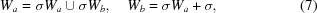
where 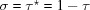 satisfies 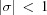. These relations are an important ingredient for the internal cocycle approach. Due to 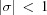, this gives rise to a contractive iterated function system, which has the windows 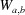 (or, more precisely, their closures) as its unique solution.

One key property, which can be employed to show that the point sets 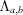 are pure point diffractive, is the fact that the 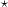-images of 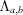 are *uniformly distributed* in the windows 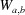, which makes it possible to translate the computation of *averaged quantities* in physical space to computations in internal space.

## 3. Shelling   

Let us discuss a simple example of an averaged quantity, the averaged shelling function for the Fibonacci point set; see Baake & Grimm (2003 ▸) for the concept and various applications to aperiodic systems. The shelling problem is related to the autocorrelation as well as to diffraction; we include it here to demonstrate, in a simple explicit example, the advantages of using internal space for this type of analysis.

For a point set, the *shelling* problem asks for the number 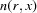 of points that lie on shells of radius *r*, taken with respect to a fixed centre *x*. For an aperiodic point set, this generally depends on the choice of the centre. The *averaged shelling* numbers 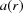 are obtained by taking the average over all choices of centres, where we limit ourselves to centres that are themselves in the point set, so 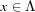. Clearly, since we are dealing with a one-dimensional point set, any shell can have at most two points, so 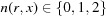 for all 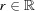, with 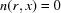 if 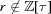, as well as 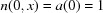. Clearly, this also implies that 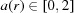 for all 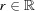, with 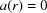 whenever 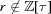.

Consider a point 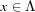 and 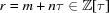. To compute 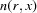, we have to check whether 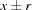 are also in the point set 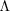. From the model set description, we know that 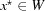, and checking whether 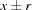 are in 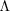 is equivalent to checking whether 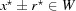. In other words, we can express 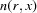 for *r* > 0 in terms of the window *W* as
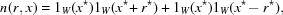
where 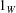 denotes the indicator (or characteristic) function of the window 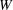, defined by
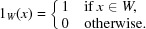
While it is possible to perform this computation for any given value of *x* and *r*, there is no simple closed formula for these coefficients.

**Figure 2:**
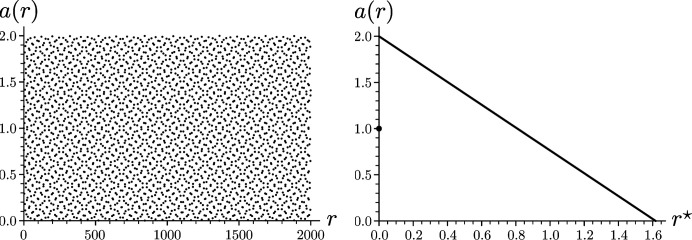
Averaged shelling numbers 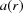 for the Fibonacci point set as a function of *r* (left) and 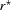 (right).

To obtain the averaged shelling number, we have to consider all 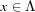 as centres, each with the same weight, which means averaging over all 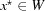. Define 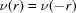 as the relative frequency to find one point of 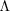 at *x* as well as one at 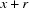, so 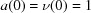 and 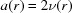 for 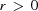, to account for the points on both sides. Now, for 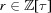, the frequency 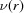 of having both 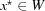 and 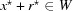 can be calculated as the overlap length between the window *W* and the shifted window 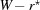, divided by the length of 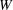, which is 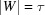. This is correct because the uniform distribution of points in the window (Moody, 2002 ▸; Baake & Grimm, 2013 ▸) implies that the frequency of any configuration is proportional to the length of the corresponding sub-window. Clearly, the length of the overlap between these two intervals is 0 whenever 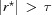, and otherwise decreases linearly with 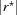, so we get
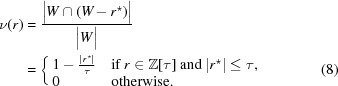
Consequently, the averaged shelling numbers for the Fibonacci point set are given by
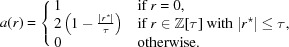
Note that 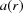, for 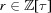, is a simple function of 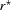, but that it behaves rather erratically if one looks at it as a function of *r*; see Fig. 2 ▸. The reason behind this observation is the total discontinuity of the 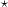-map from physical to internal space.

For the one-dimensional example at hand, the numbers 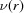 are nothing but the *relative probabilities* of finding two points at a distance *r*, and thus the (relatively normalized) *autocorrelation coefficients* of the point set 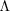. As such, they are intimately connected to the diffraction of this point set. Clearly, correlations are much easier to handle in internal space, where we can calculate them via volumes of inter­sections of windows, as we shall see shortly.

## 4. Standard approach to diffraction   

Here, we start with a brief summary of the derivation of the diffraction spectrum for the Fibonacci point set 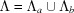, considered as a cut-and-project set 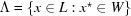 with 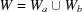. Assume that we place point scatterers of unit scattering strength at all points 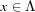, and consider the corresponding *Dirac comb*

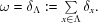
We associate to ω the autocorrelation γ = 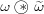, where 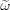 = 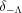 is the ‘flipped-over’ (reflected) version of ω and 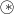 denotes volume-averaged (or Eberlein) convolution (Baake & Grimm, 2013 ▸, Section 8.8). The diffraction measure 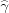 is the Fourier transform of the autocorrelation.

From the general diffraction theory for cut-and-project sets with well-behaved windows, we know that the diffraction measure of this system is a pure point measure, so consists of Bragg peaks only. These Bragg peaks are located on the projection of the entire *dual lattice*

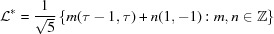
to the physical space (the first coordinate), which is 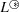 = 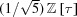. We call this set the *Fourier module* of the Fibonacci point set; it coincides with the dynamical spectrum (in additive notation) in the mathematical literature. Note that 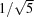 = 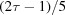, hence 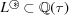, which means that the 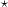-map is well defined for all 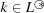. The Fourier module is a dense subset of 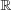, which means that the diffraction consists of Bragg peaks on a dense set in space, where the intensities are locally summable.

**Figure 3:**
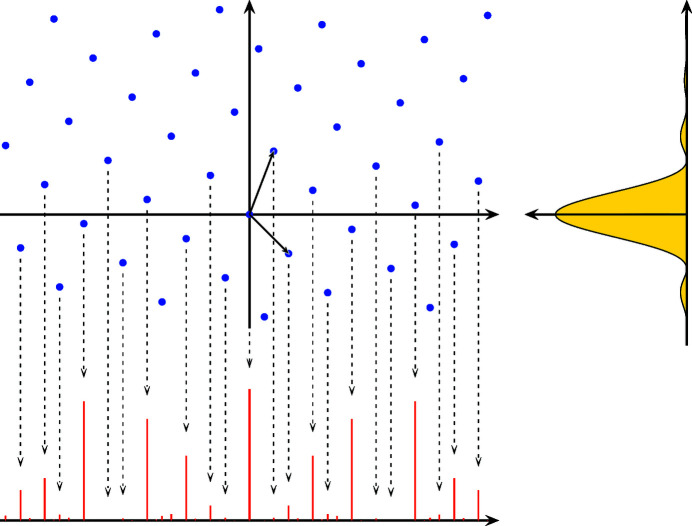
Schematic construction of the diffraction measure of the Fibonacci point set from the dual lattice 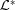 (blue dots). A point 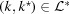 results in a Bragg peak at 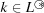 of intensity given by the value of the function on the right-hand side evaluated at 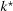. Note that some Bragg peaks may be extinct, if the intensity function vanishes at 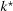.

The diffraction measure is thus the countable sum
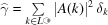
where the diffraction amplitudes, or *Fourier–Bohr* (FB) coefficients, are given by the general formula
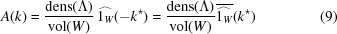
for all 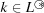, and vanish otherwise. Here,
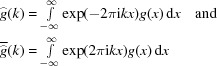
denote the *Fourier* and the *inverse Fourier transform* of a real-valued 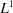-function *g*. With 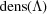 = 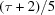 and 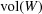 = |*W*| = τ, equation (9) evaluates to
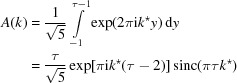
where 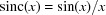. Hence, the diffraction intensities are
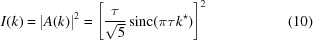
for all 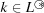, and 0 otherwise. This is illustrated in Fig. 3 ▸. Note that 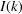 can vanish for some 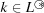, in which case we talk of an *extinction* of the Bragg peak. For the Fibonacci system, this may happen for specific choices of the scattering strengths (such as in our simple case, where we chose them to be 1 for all points in 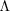). However, for a generic choice of weights [see (11) below], there will be no extinctions, and we will have a Bragg peak for all 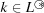.

The corresponding autocorrelation measure γ can be expressed in terms of the (dimensionless) *pair correlation coefficients*

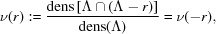
which are positive for all 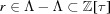 and vanish for all other distances *r*. These are precisely the coefficients we defined in equation (8) to compute the shelling numbers. The link between the two expressions is provided by the 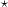-map and the uniform distribution of 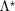 in the window 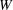. In terms of these pair correlation coefficients, the autocorrelation measure is
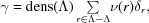
which is a pure point measure supported on the difference set 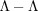.

More generally, we may associate two different, in general complex, scattering strengths 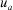 and 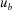 to the points in 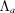 and 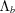, respectively, and consider the weighted Dirac comb 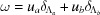. In this case, the diffraction intensity for all wavenumbers 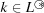 is given by the superposition
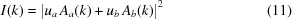
of the corresponding FB amplitudes
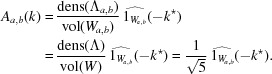
The corresponding autocorrelation measure can once more be expressed in terms of pair correlation functions, now distinguishing points in 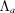 and 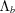,
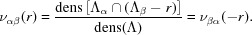
These coefficients are positive for all 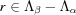 and vanish otherwise, and in particular satisfy the relation 
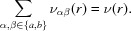



The relation (9) between the FB coefficients and the Fourier transform of the compact windows holds for any regular model set, which is a cut-and-project set with some ‘niceness’ constraint on the window; see Theorem 9.4 in Baake & Grimm (2013 ▸) for details. While this works well for many of the nice examples with polygonal windows, it becomes practically impossible to compute the FB coefficients in this way if the windows are compact sets with fractal boundaries. Such windows naturally arise for cut-and-project sets which also possess an inflation symmetry. Indeed, some of the structure models of icosahedral quasicrystals, see Takakura *et al.* (2007 ▸) for an example, feature experimentally determined windows whose shapes may indicate first steps of a fractal construction of the boundary.

Let us therefore explain a different approach that will permit an efficient computation of the diffraction also for such, more complicated, situations.

## 5. Renormalization and internal cocycle   

Let us reconsider our motivating example, the Fibonacci point sets 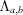 of equation (6). We will use both their inflation structure and their description as cut-and-project sets. Here, we make use of the iteration (5) and the corresponding relation (7) for the windows (or, more precisely, the closure of the windows). This inflation structure induces the following relation between the characteristic functions of the windows,
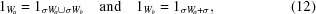
where we again set σ = 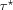 = 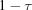. Since the (closed) windows only share at most boundary points, we observe that 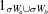 = 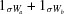 holds as an equality of 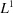-functions. Now, we can apply the Fourier transform, which yields the relations
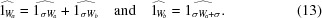
These equations capture the action of the inflation in internal space in terms of functional equations for the Fourier transform of the windows, which in turn determine the diffraction. In what follows, it turns out to be more convenient to work with the inverse Fourier transform. Note that, by an elementary change of variable calculation in the Fourier integral, one has
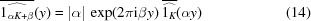
for arbitrary 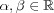 with 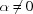 and any compact set 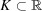. This can be used to express the functions in (13) with σ-scaled and shifted windows in terms of the indicator functions of the original windows.

Indeed, defining
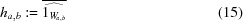
for the two functions involving the original windows, and using equation (14), we can rewrite equation (13) as
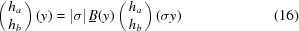
with the matrix
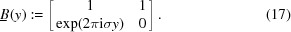
The matrix 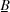 is obtained by first taking the 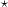-map of the set-valued displacement matrix *T* of equation (4) and then its inverse Fourier transform. For this reason, 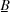 is called the *internal Fourier matrix* (Baake & Grimm, 2019*b* ▸), to distinguish it from the Fourier matrix of the renormalization approach in physical space (Baake & Gähler, 2016 ▸; Baake, Frank *et al.*, 2019 ▸); see Bufetov & Solomyak (2018 ▸, 2020 ▸) for various extensions with more flexibility in the choice of the interval lengths.

In Dirac notation, we set 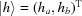, which satisfies 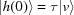 with the right eigenvector 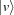 of the substitution matrix *M* from equation (2). Applying the iteration (16) *n* times then gives
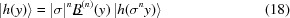
where
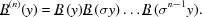
In particular, these matrices satisfy 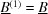 and 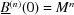 for all 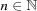, where *M* is the substitution matrix from equation (1), as well as the relations
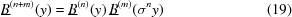
for any 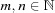. Note that 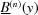 defines a matrix cocycle, called the *internal cocycle*, which is related to the usual inflation cocycle (in physical space) by an application of the 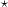-map to the displacement matrices of the powers of the inflation rule; compare Baake, Gähler & Mañibo (2019 ▸), Baake & Grimm (2019*b* ▸), and see Bufetov & Solomyak (2018 ▸, 2020 ▸) for a similar approach. Note also that 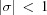, which means that 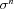 approaches 0 exponentially fast as 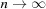. We can exploit this exponential convergence to efficiently compute the diffraction amplitudes, which are proportional to the elements of the vector 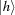.

Considering the limit as 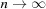 in equation (18), one can show that
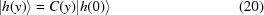
with
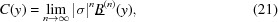
which exists pointwise for every 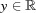. In fact, one has compact convergence, which implies that 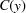 is continuous (Baake & Grimm, 2019*b* ▸, Theorem 4.6 and Corollary 4.7). Clearly, since 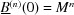, we have 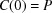 with the projector 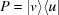 from equation (3).

Using equation (19) with 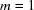 and letting 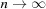, one obtains
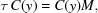
since 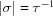. This relation implies that each row of 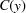 is a multiple of the left eigenvector 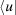 of the substitution matrix *M* from equation (2), so there is a vector-valued function 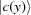 such that
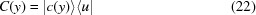
holds with 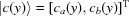, where we have 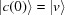.

From equations (20) and (22), we obtain
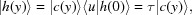
and the inverse Fourier transforms of the windows from equation (15) are thus encoded in the matrix *C*.

For the Fibonacci case, we can calculate 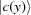 by taking the Fourier transforms of the known windows 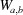 to obtain
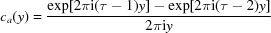
and 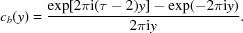
Note that these functions never vanish simultaneously, so 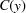 is always a matrix of rank 1. However, taking the Fourier transform of the windows takes us essentially back to the standard approach.

The main benefit of the internal cocycle approach is that it applies in other situations, where no explicit calculation of the (inverse) Fourier transform of the windows is feasible. This is achieved via *approximating* 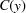 by 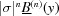 for a sufficiently large *n*, such that 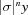 is small and 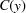 is approximated sufficiently well. This works because the (closed) windows are compact sets, so that their (inverse) Fourier transforms are continuous functions. The convergence of this approximation is exponentially fast. We refer readers to Baake & Grimm (2019*b* ▸) for further details and an extension of the cocycle approach to more general inflation systems, and to Baake & Grimm (2020 ▸) for a planar example.

From the general formula (9) for regular model sets, the FB amplitudes are
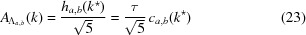
for 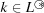. So, the relevant input is the knowledge of the Fourier module, which determines where the Bragg peaks are located. Then, one can approximate *C* by evaluating the matrix product in equation (21), for any chosen 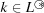, at 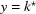 and with a sufficiently large *n*. In what follows, numerical calculations and illustrations are based on this cocycle approach due to its superior speed and accuracy in the presence of complex windows.

## 6. Fractally bounded windows   

The internal cocycle approach of Section 5 was first applied to a ternary inflation tiling with the smallest Pisot–Vijayaraghavan (PV) number (also known as the ‘plastic number’) as its inflation multiplier (Baake & Grimm, 2020 ▸). In the cut-and-project description, the internal space of this one-dimensional tiling is two-dimensional, and the windows are *Rauzy fractals* (Pytheas Fogg, 2002 ▸). This means that the windows are still topologically regular, so each window is the closure of its interior, but they have a fractal boundary of zero Lebesgue measure. Consequently, the general diffraction result for model sets still applies, and the diffraction is given by the Fourier transform of the windows as described above. In turn, this means that the internal cocycle approach applies and can be used to compute the Fourier transforms and the diffraction intensities for such tilings; see Baake & Grimm (2020 ▸) for details.

**Figure 4:**
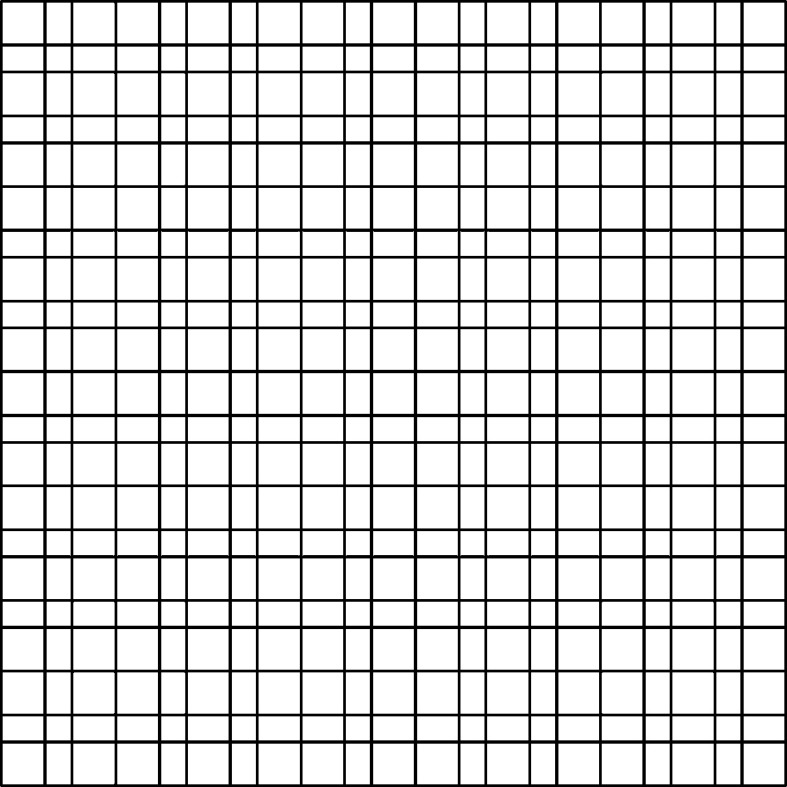
Patch of the square Fibonacci tiling.

Here, we discuss examples of planar projection tilings with fractally bounded windows, which are based on *direct product variations* (DPVs) (Sadun, 2008 ▸; Frank, 2015 ▸) of Fibonacci systems, as recently described by Baake *et al.* (2021 ▸). Clearly, if one considers a direct product structure based on the Fibonacci tiling, one obtains a tiling of the plane, called the *square Fibonacci tiling*. This tiling has been used as a toy model for the study of electronic properties (Lifshitz, 2002 ▸; Even-Dar Mandel & Lifshitz, 2008 ▸; Damanik & Gorodetski, 2018 ▸), but has been observed experimentally to form in a molecular overlayer on a twofold surface of an icosahedreal quasicrystal (Coates *et al.*, 2018 ▸). It is built from four prototiles, a large square of edge length τ, a small square of edge length 1, and two rectangles with a long (τ) and a short (1) edge; see Fig. 4 ▸.

As a direct product of inflation tilings, this two-dimensional square Fibonacci tiling also possesses an inflation rule, which takes the form[Chem scheme1] 
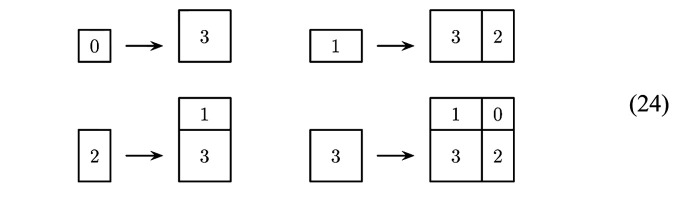


where we labelled the small and large squares by 0 and 3, and the two rectangles by 1 and 2, respectively. A DPV is now obtained by modifying these rules while keeping the stone inflation character intact, thus probing the ideas of Clark & Sadun (2006 ▸) into a slightly different direction. Clearly, there are two possibilities to rearrange the images of the rectangles by swapping the two tiles, and a close inspection shows that there are altogether 12 ways of rearranging the image of the large square. This means that there are 48 distinct inflation rules in total, which all share these prototiles and the same inflation matrix.

**Figure 5:**
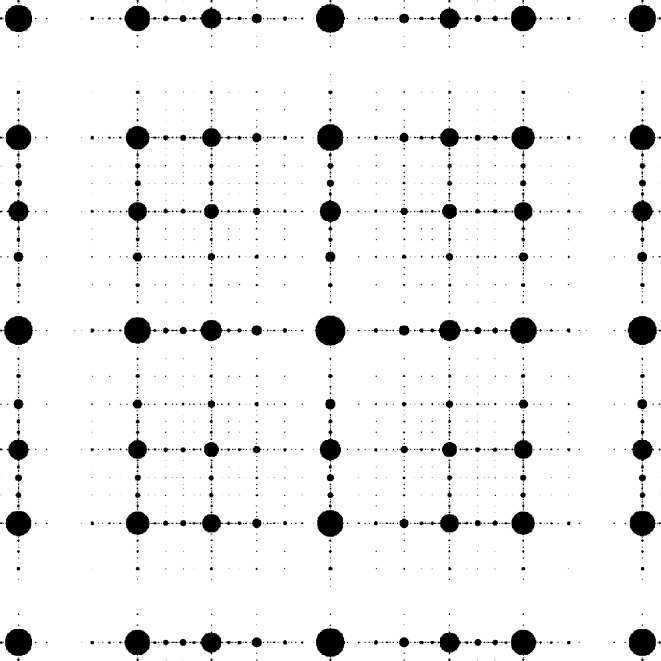
Central part of the diffraction image of the square Fibonacci tiling.

Due to the direct product structure, the square Fibonacci tiling clearly possesses a cut-and-project description. The windows for the four prototiles are obtained as products of the original windows. The product structure thus extends to the diffraction measure, which is supported on the Fourier module
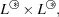
where 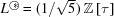 is the Fourier module of the one-dimensional Fibonacci tiling. The diffraction amplitudes are also given by products of those for the one-dimensional system, and are thus easy to compute. An illustration of the diffraction pattern is shown in Fig. 5 ▸. Here, Bragg peaks are represented by discs, centred at the position of the peak, with areas proportional to their intensities.

It turns out that *all* 48 DPV inflation tilings are regular model sets, and hence are pure point diffractive; see Theorem 5.2 in Baake *et al.* (2021 ▸). They all share the same Fourier module, 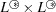. This implies that the Bragg peaks are always located at the same positions (where we disregard possible extinctions). However, their intensities are determined by the Fourier transform of the windows, and it turns out that the windows of these DPVs can differ substantially.

In particular, 20 of these DPVs possess windows of Rauzy fractal type, of which there are three different types, called ‘castle’, ‘cross’ and ‘island’ by Baake *et al.* (2021 ▸). They have different fractal dimension of the window boundaries, which are approximately 1.875, 1.756 and 1.561, respectively. As the dimensions are all smaller than two, is it obvious that these boundaries have zero Lebesgue measure.

In what follows, we are going to illustrate some properties of these DPVs with three examples, one for each of these fractally bounded window types. The inflation rules for the three examples have the same images for the small square (tile 0) and both rectangles (tiles 1 and 2) as the square Fibonacci rule of equation (24), and thus only differ in the image of the large square (tile 3). For a discussion of the complete set of 48 DPVs, we refer readers to Baake *et al.* (2021 ▸).

**Figure 6:**
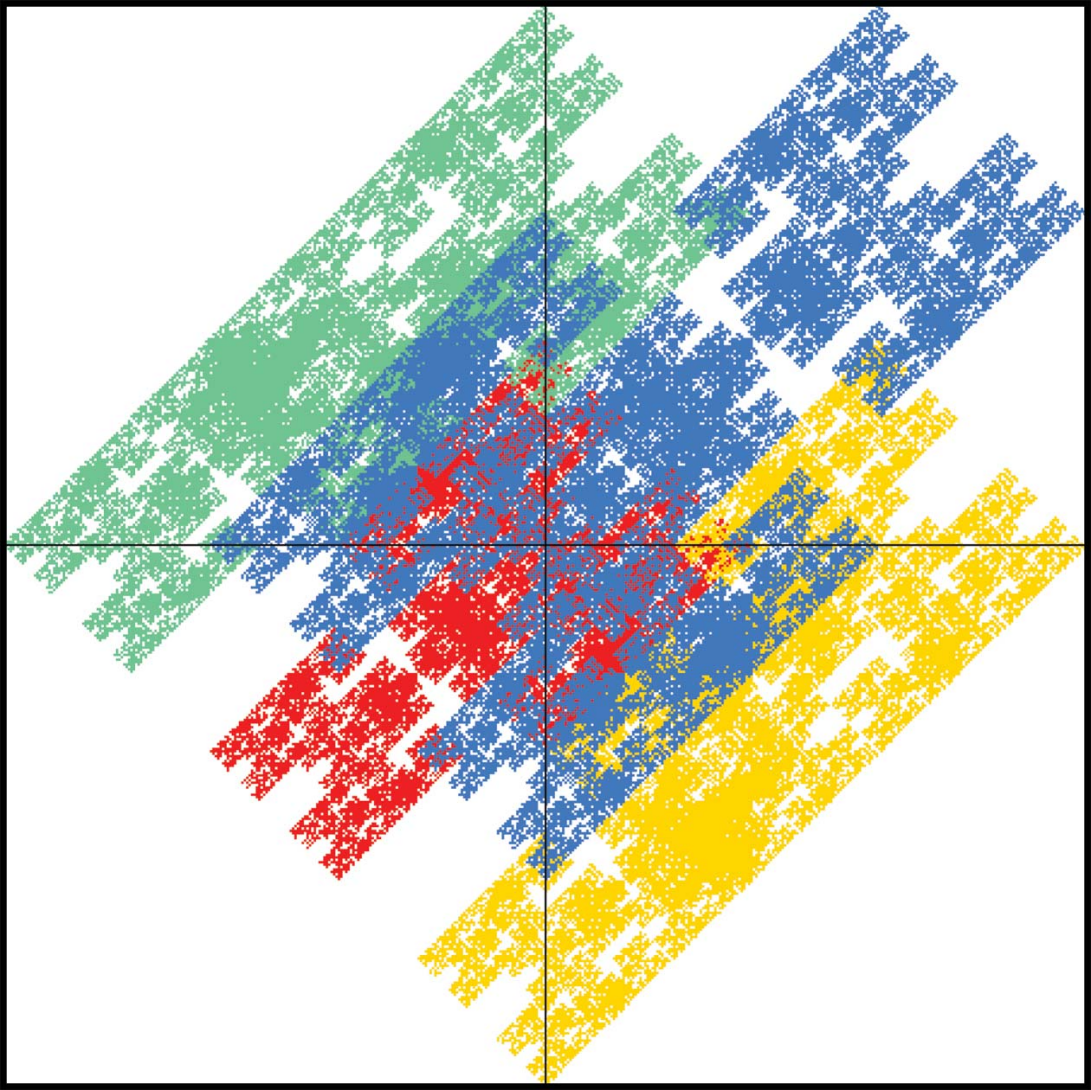
Castle-type window for the DPV (25). The windows for the four types of tiles are distinguished by colour, namely red (0), yellow (1), green (2) and blue (3). The outer box marks the square 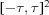, with the coordinate axes indicated as well.

**Figure 7:**
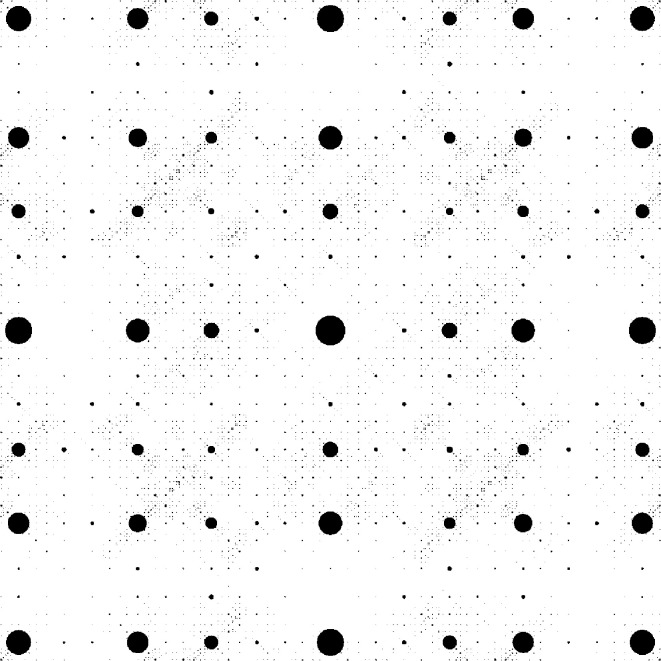
Diffraction image of the DPV (25).

For the castle-type windows of Fig. 6 ▸, we use the inflation[Chem scheme2] 
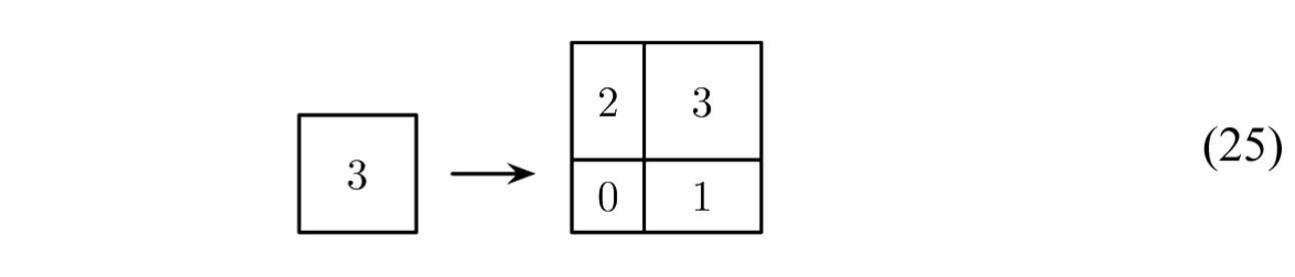


for the large square. Note that this rule dissects the inflated large square such that there is a reflection symmetry along the main diagonal, which will be reflected in a symmetry of the tiling (which maps the squares onto themselves and interchanges the rectangles). This is also apparent for the windows in Fig. 6 ▸. The windows for the large and small squares are mapped onto themselves under reflection at the main diagonal, while the windows for the rectangular tiles are interchanged. The diffraction pattern also respects this symmetry; see Fig. 7 ▸.

**Figure 8:**
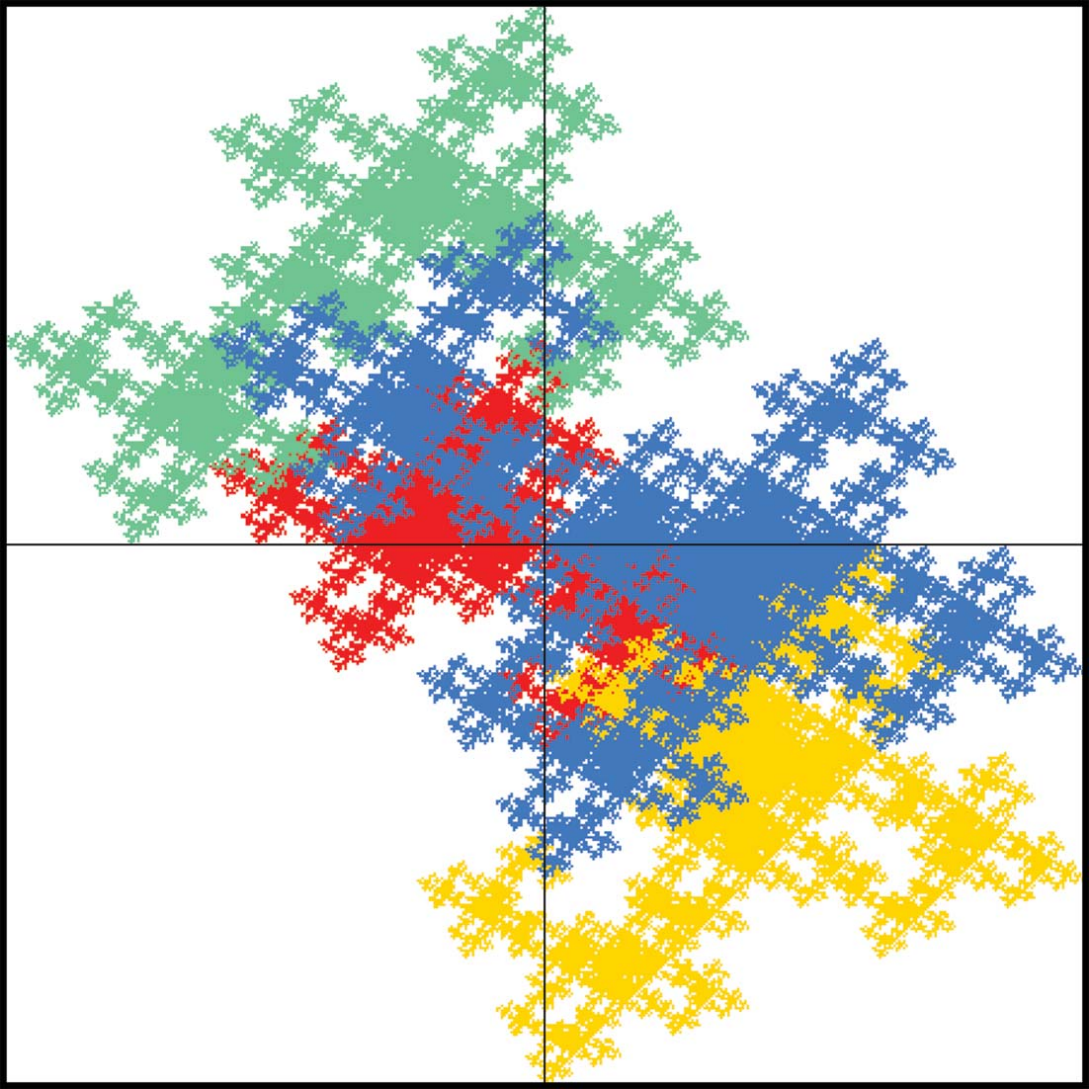
Cross-type window for the DPV (26).

**Figure 9:**
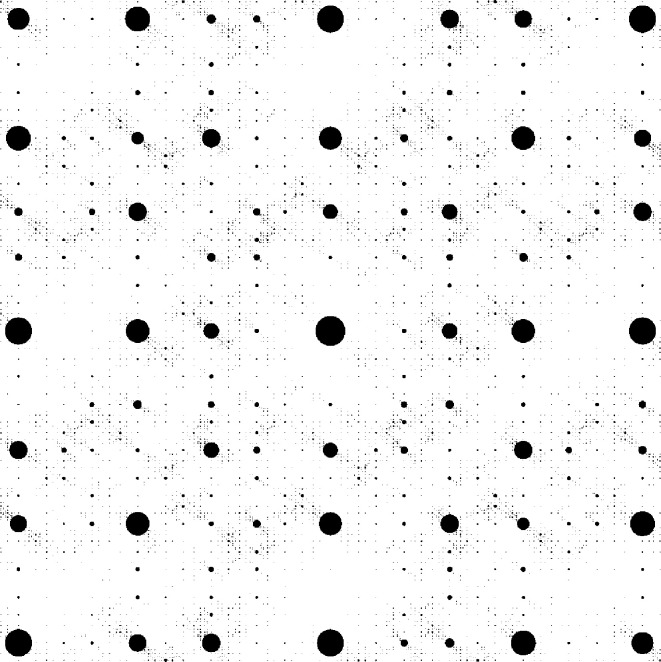
Diffraction image of the DPV (26).

For the cross-type windows, the inflation of the large square is given by[Chem scheme3] 
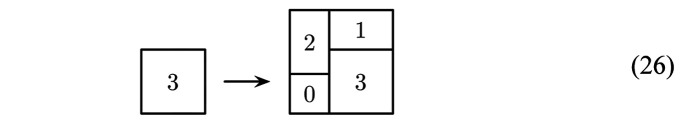


which, in contrast to the previous example, has no reflection symmetry. Consequently, neither the windows shown in Fig. 8 ▸ nor the diffraction image illustrated in Fig. 9 ▸ have any reflection symmetry.

**Figure 10:**
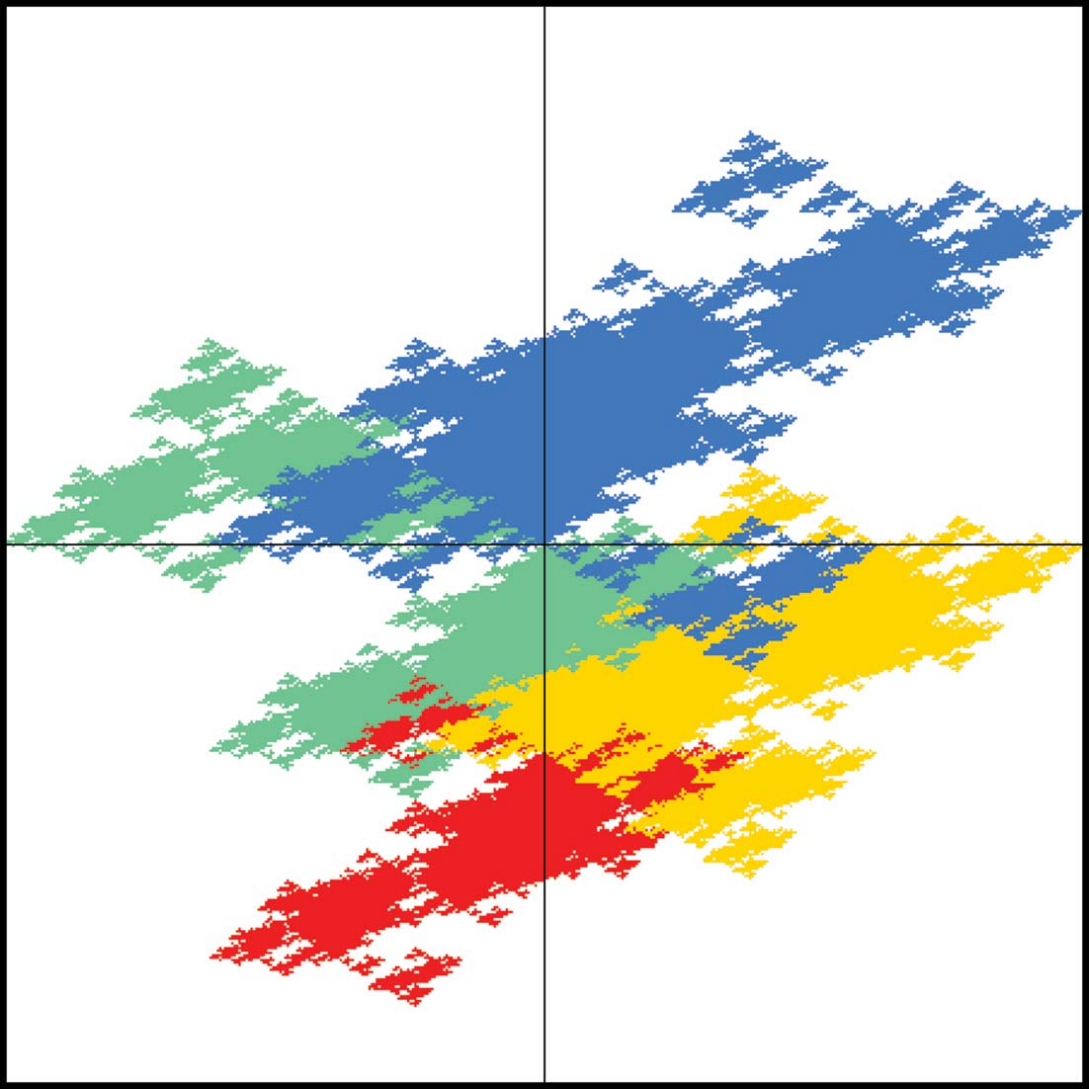
Island-type window for the DPV (27).

**Figure 11:**
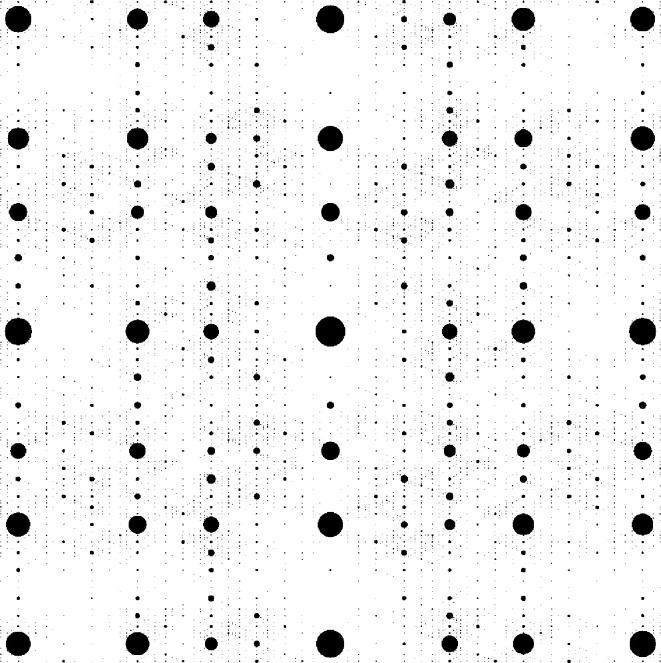
Diffraction image of the DPV (27).

The same is true for the final example with the island-type window shown in Fig. 10 ▸. This corresponds to the inflation[Chem scheme4] 
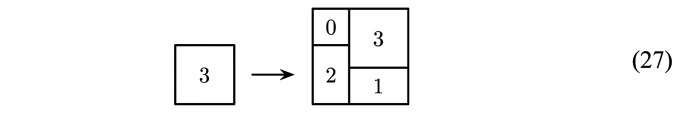


of the large square tile. The corresponding diffraction pattern is illustrated in Fig. 11 ▸.

Comparing the diffraction patterns of Figs. 7 ▸, 9 ▸ and 11 ▸ with those of the square Fibonacci tiling shown in Fig. 5 ▸, we note that the strongest peaks are almost unchanged, while the intensities of the weaker peaks show some intriguing behaviour. The reason for this behaviour is that all three model sets are subsets of a common Meyer set, and the so-called ∊-dual characters of the difference set of this Meyer set, for small ∊, always give rise to high-intensity Bragg peaks; see Strungaru (2013 ▸) for details. This is the reason why the strongest peaks stay almost the same.

For the fractally bounded windows, one generally sees more peaks, which is due to the larger spread of the window in internal space, and the slower asymptotic decay of the Fourier transform of the window (as 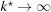). With limited resolution, some of the intensity distributions on these peaks could resemble continuous components, so might potentially be mistaken as such in experiments.

## 7. Diffraction and hyperuniformity   

The discovery of quasicrystals highlighted the lack of a clear definition of the concept of *order*. In crystallography, diffraction is the main tool to detect long-range order, and a pure point diffraction is generally associated with an ordered, (quasi)crystalline structure, while absolutely continuous diffraction is typically seen as an indication of random disorder [but see Frank (2003 ▸), Baake & Grimm (2009 ▸), Chan & Grimm (2017 ▸), Chan *et al.* (2018 ▸) for examples of deterministic structures that show absolutely continuous diffraction]. Here, we briefly discuss a related concept that has recently gained popularity.

From the original idea of using the degree of ‘(hyper)uniformity’ in density fluctuations in many-particle systems (Torquato & Stillinger, 2003 ▸; Brauchart *et al.*, 2019 ▸, 2018 ▸) to characterize their order, the *scaling* behaviour of the total diffraction intensity near the origin has emerged as a possible measure to capture long-distance correlations. As far as aperiodic structures are concerned, there are in fact a number of early, partly heuristic, results in the literature (Luck, 1993 ▸; Aubry *et al.*, 1988 ▸; Godrèche & Luck, 1990 ▸). These have recently been reformulated and extended (Oğuz *et al.*, 2017 ▸, 2019 ▸) and rigorously established (Baake & Grimm, 2019*a* ▸), using exact renormalization relations for primitive inflation rules (Baake, Frank *et al.*, 2019 ▸; Baake & Gähler, 2016 ▸; Mañibo, 2017 ▸, 2019 ▸; Baake, Gähler & Mañibo, 2019 ▸; Baake *et al.*, 2018 ▸); see also Fuchs *et al.* (2019 ▸) for results for some planar aperiodic tilings.

For the investigation of scaling properties, we follow the existing literature and define
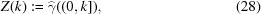
which is a modified version of the *distribution function* of the diffraction measure. Here, 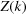 is the total diffraction intensity in the half-open interval 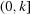, and thus ignores the central peak. Due to the point reflection symmetry of 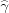 with respect to the origin, this quantity can also be expressed as
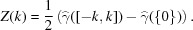
The interest in the scaling of 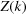 as 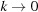 is motivated by the intuition that the small-*k* behaviour of the diffraction measure probes the long-wavelength fluctuations in the structure. As the latter is related to the variance in the distribution of patches, it can serve as an indicator for the degree of uniformity of the structure (Torquato & Stillinger, 2003 ▸). It is obvious that any periodic structure leads to 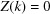 for all sufficiently small wavenumbers *k*.

Here, we review the result for variants of the one-dimensional Fibonacci model sets considered above, where we now allow for changes of the windows. For a general discussion of this approach and more examples of systems with different types of diffraction, we refer readers to Baake & Grimm (2019*a* ▸) and references therein.

Let us look at the diffraction for a cut-and-project set with the same setup as the Fibonacci tiling considered in Section 4, but with the window *W* replaced by an arbitrary finite interval of length *s*. Note that these tilings, in general, do *not* possess an inflation symmetry. Nevertheless, the diffraction intensity is still of the form (10), but now featuring the interval length *s*, and is given by
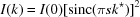
for all 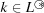. Now, consider a sequence of positions 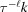 with 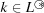 and 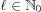. Since we have 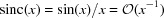
as 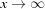, it follows that 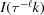 = 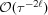 as 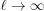.

Consequently, the sum of intensities along the series of peaks,
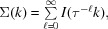
satisfies the asymptotic behaviour
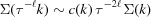
as 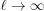, where it can be shown that 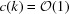 (Baake & Grimm, 2019*a* ▸). Expressing 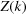 in terms of these sums gives
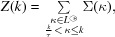
which implies the asymptotic behaviour
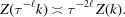
This leads to a power-law scaling behaviour of the form 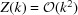 as 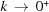.

This generic result remains true if we choose a window which corresponds to a tiling with inflation symmetry, which requires the window to be an interval of length 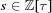. This obviously holds for our original Fibonacci window *W* of length τ. However, one gets a stronger result for this case (Baake & Grimm, 2019*a* ▸; Oğuz *et al.*, 2017 ▸), as we shall now recall.

Choosing 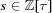 means *s* = 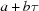 with 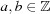. For 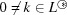, set *k* = 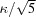 with κ = 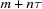 for some 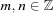, excluding *m* = *n* = 0. Applying the 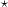-map then gives
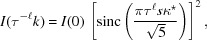
with 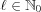.

Now, denote by 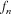 with 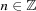 the Fibonacci numbers defined by 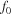 = 0, 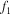 = 1 and the recursion 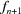 = 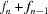. They satisfy the well-known formula
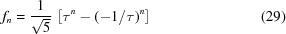
for all 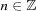. Using this relation, we obtain
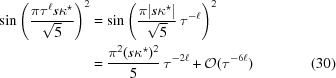
as 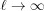. Here, the first step follows by using equation (29) to replace 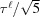 and then reducing the argument via the relation
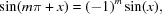
which holds for all 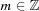 and 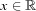. This is possible because all Fibonacci numbers are integers. The second step then uses the Taylor approximation sin(*x*) = 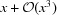 for small values of *x*.

**Figure 12:**
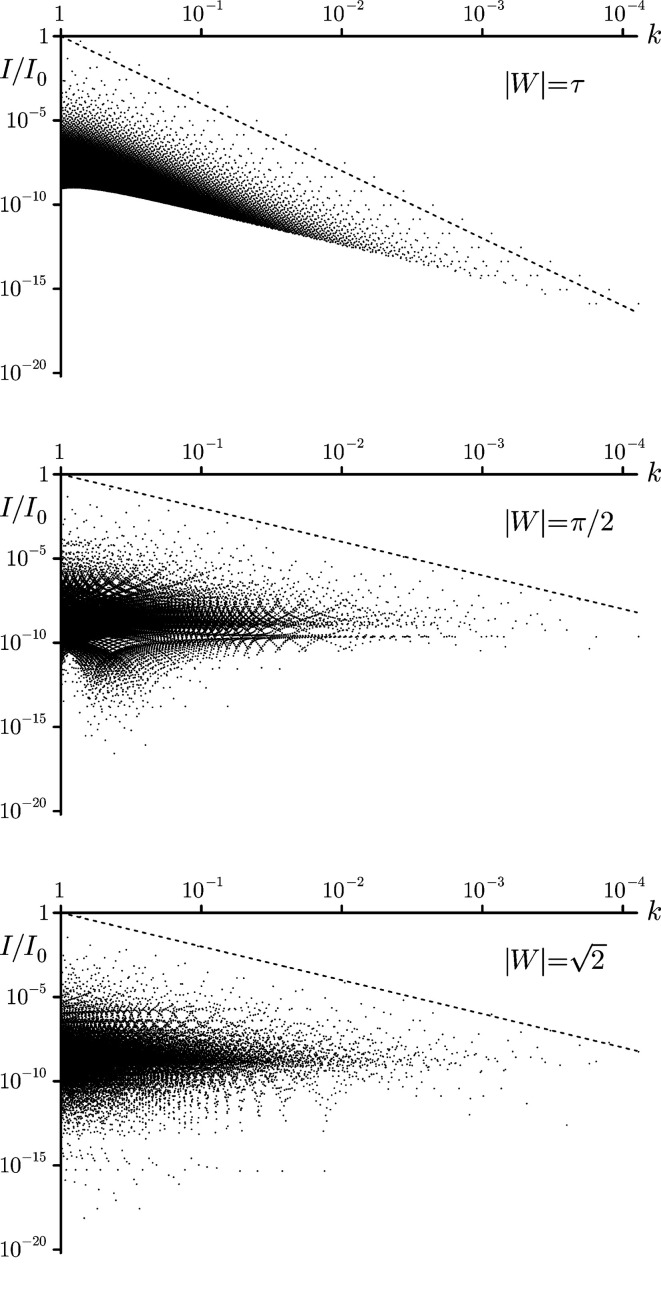
Double logarithmic plot of the intensity ratio 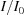 of Bragg peaks located at *k* = 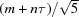 with 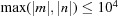, where 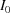 = *I*(0), for windows *W* of different lengths. The dashed line corresponds to 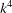 for length 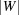 = τ (top) and to 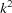 for the other two cases.

Now, the same argument as above implies the asymptotic behaviour
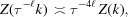
and hence *Z*(*k*) = 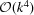. This result means that, for inflation-invariant projection sets, the distribution function 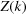 of the diffraction intensity vanishes like 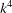 as 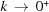, while, in the generic case, we find a 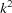-behaviour. This example illustrates that the behaviour of the diffraction intensity near 0 can pick up non-trivial aspects of order in this system. This is illustrated for some cases in Fig. 12 ▸.

Our discussion above may appear quite special, in the sense that we chose all scattering strengths to be equal. However, since we are only interested in the scaling behaviour near the origin, this is in fact no restriction, because the scaling law is unaffected by changing the scattering strengths (as the length of the total window falls into 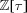 if and only if the lengths of the sub-windows do). This simultaneously points to a strength and a weakness of this quantity as a measure of order. On the one hand, the scaling behaviour can detect and distinguish the order in the spatial arrangement of atoms irrespective of the scattering strengths of the atoms; on the other hand, it cannot provide any information on the distribution of different scatterers. For the latter, the knowledge of the intensities of the Bragg peaks is required.

Let us briefly comment on the scaling behaviour for other prominent examples of aperiodic order discussed by Baake & Grimm (2019*a* ▸). For noble means inflations, we observe the same 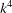-scaling as for the Fibonacci tiling. The period doubling sequence, which is limit periodic, shows 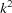-scaling, and a range of scaling exponents is accessible for substitutions of more than two letters. For the Thue–Morse sequence, which is the paradigm of an inflation structure with singular continuous diffraction, we do not obtain a power law, but an exponential scaling behaviour which decays faster than any power; see also Baake, Gohlke *et al.* (2019 ▸) for more on the scaling of the spectrum for this system. Finally, the Rudin–Shapiro sequence, which has an absolutely continuous spectrum, shows a linear scaling behaviour, due to the constant density of its diffraction measure.

## References

[bb1] Akiyama, S. & Arnoux, P. (2020). Editors. *Tiling Dynamical Systems: Substitutions and Beyond.* Berlin: Springer.

[bb2] Akiyama, S., Barge, M., Berthé, V., Lee, J.-Y. & Siegel, A. (2015). *Mathematics of Aperiodic Order*, edited by J. Kellendonk, D. Lenz & J. Savinien, pp. 33–72. Basel: Birkhäuser.

[bb3] Allouche, J.-P. & Shallit, J. (2003). *Automatic Sequences.* Cambridge University Press.

[bb4] Aubry, S., Godrèche, C. & Luck, J. M. (1988). *J. Stat. Phys.* **51**, 1033–1075.

[bb5] Baake, M., Coons, M. & Mañibo, N. (2020). *From Analysis to Visualization: JBCC 2017*, edited by D. Bailey, N. S. Borwein, R. P. Brent, R. S. Burachik, J.-A. H. Osborn, B. Sims & Q. J. Zhu, pp. 303–322. Cham: Springer.

[bb6] Baake, M., Ecija, D. & Grimm, U. (2016). *Z. Kristallogr.* **231**, 507–515.

[bb7] Baake, M., Frank, N. P. & Grimm, U. (2021). *Stoch. Dyn.* **21**, 214001.

[bb8] Baake, M., Frank, N. P., Grimm, U. & Robinson, E. A. (2019). *Stud. Math.* **247**, 109–154.

[bb9] Baake, M. & Gähler, F. (2016). *Topol. Appl.* **205**, 4–27.

[bb10] Baake, M., Gähler, F. & Grimm, U. (2013). *J. Integer Seq.* **16**, 13.2.14.

[bb11] Baake, M., Gähler, F. & Mañibo, N. (2019). *Commun. Math. Phys.* **370**, 591–635.

[bb12] Baake, M., Gohlke, P., Kesseböhmer, M. & Schindler, T. (2019). *Discrete Cont. Dyn. Syst. A*, **39**, 4157–4185.

[bb13] Baake, M. & Grimm, U. (2003). *Discrete Comput. Geom.* **30**, 573–589.

[bb14] Baake, M. & Grimm, U. (2009). *Phys. Rev. B*, **79**, 020203.

[bb15] Baake, M. & Grimm, U. (2012). *Chem. Soc. Rev.* **41**, 6821–6843. 10.1039/c2cs35120j 22797147

[bb16] Baake, M. & Grimm, U. (2013). *Aperiodic Order*, Vol. 1, *A Mathematical Invitation*. Cambridge University Press.

[bb17] Baake, M. & Grimm, U. (2019*a*). *J. Stat. Mech.* **2019**, 054003.

[bb18] Baake, M. & Grimm, U. (2019*b*). arXiv:1907.11012.

[bb19] Baake, M. & Grimm, U. (2020). *J. Phys. Conf. Ser.* **1458**, 012006.

[bb20] Baake, M., Grimm, U. & Mañibo, N. (2018). *Lett. Math. Phys.* **108**, 1783–1805.

[bb21] Baake, M., Huck, C. & Strungaru, N. (2017). *Indag. Math.* **28**, 3–31.

[bb22] Baake, M. & Zeiner, P. (2017). *Aperiodic Order* Vol. 2, *Crystallography and Almost Periodicity*, edited by M. Baake & U. Grimm, pp. 73–172. Cambridge University Press.

[bb23] Bartlett, A. (2018). *Ergod. Th. Dyn. Syst.* **38**, 1289–1341.

[bb24] Berlinkov, A. & Solomyak, B. (2019). *Ergod. Th. Dyn. Syst.* **39**, 2384–2402.

[bb25] Brauchart, J. S., Grabner, P. J. & Kusner, W. (2019). *Constr. Approx*, **50**, 45–61.

[bb26] Brauchart, J. S., Grabner, P. J., Kusner, W. B. & Ziefle, J. (2018). arXiv:1809.02645.

[bb27] Bufetov, A. & Solomyak, B. (2018). arXiv:1802.04783.

[bb28] Bufetov, A. & Solomyak, B. (2020). arXiv:2003.11287.

[bb29] Chan, L. & Grimm, U. (2017). *Adv. Appl. Math.* **87**, 16–23.

[bb30] Chan, L., Grimm, U. & Short, I. (2018). *Indag. Math.* **29**, 1072–1086.

[bb31] Clark, A. & Sadun, L. (2006). *Ergod. Th. Dyn. Syst.* **26**, 69–86.

[bb32] Coates, S., Smerdon, J. A., McGrath, R. & Sharma, H. R. (2018). *Nat. Commun.* **9**, 3435. 10.1038/s41467-018-05950-7 PMC6109137 30143631

[bb33] Damanik, D. & Gorodetski, A. (2018). *J. Spectr. Theory*, **8**, 1487–1507.

[bb34] Even-Dar Mandel, S. & Lifshitz, R. (2008). *Philos. Mag.* **88**, 2261–2273.

[bb35] Frank, N. P. (2003). *Ergod. Th. Dyn. Syst.* **23**, 519–532.

[bb36] Frank, N. P. (2015). *Mathematics of Aperiodic Order*, edited by J. Kellendonk, D. Lenz & J. Savinien, pp. 223–257. Basel: Birkhäuser.

[bb37] Frettlöh, D. (2017). *Aperiodic Order*, Vol. 2, *Crystallography and Almost Periodicity*, edited by M. Baake & U. Grimm, pp. 1–37. Cambridge University Press.

[bb38] Fuchs, J.-N., Mosseri, R. & Vidal, J. (2019). *Phys. Rev. B*, **100**, 125118.

[bb39] Godrèche, C. & Luck, J. M. (1990). *J. Phys. A Math. Gen.* **23**, 3769–3797.

[bb40] Grimm, U. (2015). *Acta Cryst.* B**71**, 258–274. 10.1107/S2052520615008409 26027002

[bb41] Hof, A. (1995). *Commun. Math. Phys.* **169**, 25–43.

[bb42] Kellendonk, J., Lenz, D. & Savinien, J. (2015). Editors. *Mathematics of Aperiodic Order.* Basel: Birkhäuser.

[bb43] Lifshitz, R. (2002). *J. Alloys Compd.* **342**, 186–190.

[bb44] Luck, J. M. (1993). *Europhys. Lett.* **24**, 359–364.

[bb46] Mañibo, C. N. (2019). *Lyapunov Exponents in the Spectral Theory of Primitive Inflation Systems*. PhD thesis, Bielefeld University, Germany. urn:nbn:de:0070-pub-29359727.

[bb45] Mañibo, N. (2017). *J. Math. Phys.* **58**, 113504.

[bb47] Moody, R. V. (2000). *From Quasicrystals to More Complex Systems*, edited by F. Axel, F. Dénoyer & J. P. Gazeau, pp. 145–166. Berlin: Springer.

[bb48] Moody, R. V. (2002). *Can. Math. Bull.* **45**, 123–130.

[bb49] Oğuz, E. C., Socolar, J. E. S., Steinhardt, P. J. & Torquato, S. (2017). *Phys. Rev. B*, **95**, 054119.

[bb50] Oğuz, E. C., Socolar, J. E. S., Steinhardt, P. J. & Torquato, S. (2019). *Acta Cryst.* A**75**, 3–13. 10.1107/S2053273318015528 PMC6302933 30575579

[bb51] Pytheas Fogg, N. (2002). *Substitutions in Dynamics, Arithmetics and Combinatorics*, LNM 1794. Berlin: Springer.

[bb52] Queffélec, M. (2010). *Substitution Dynamical Systems – Spectral Analysis*, 2nd ed., LNM 1294. Berlin: Springer.

[bb53] Richard, C. & Strungaru, N. (2017*a*). *J. Phys. A: Math. Theor.* **50**, 154003.

[bb54] Richard, C. & Strungaru, N. (2017*b*). *Ann. Henri Poincaré*, **18**, 3903–3931.

[bb55] Sadun, L. (2008). *Topology of Tiling Spaces.* Providence: American Mathematical Society.

[bb56] Schlottmann, M. (2000). *Directions in Mathematical Quasicrystals*, edited by M. Baake & R. V. Moody, pp. 143–159. Providence: American Mathematical Society.

[bb57] Shechtman, D., Blech, I., Gratias, D. & Cahn, J. W. (1984). *Phys. Rev. Lett.* **53**, 1951–1953.

[bb58] Solomyak, B. (1997). *Ergod. Th. Dyn. Syst.* **17**, 695–738.

[bb59] Strungaru, N. (2013). *Can. J. Math.* **65**, 675–701.

[bb60] Strungaru, N. (2017). *Aperiodic Order*, Vol. 2, *Crystallography and Almost Periodicity*, edited by M. Baake & U. Grimm, pp. 271–342. Cambridge University Press.

[bb61] Strungaru, N. (2020). *J. Funct. Anal.* **278**, 108404.

[bb62] Takakura, H., Gómez, C. P., Yamamoto, A., de Boissieu, M. & Tsai, A. P. (2007). *Nat. Mater.* **6**, 53–63. 10.1038/nmat1799 17160006

[bb63] Torquato, S. & Stillinger, F. H. (2003). *Phys. Rev. E*, **68**, 041113. 10.1103/PhysRevE.68.041113 14682929

